# Genomics at Belyaev conference – 2017

**DOI:** 10.1186/s12864-018-4476-5

**Published:** 2018-02-09

**Authors:** Yuriy L. Orlov, Ancha V. Baranova, Ralf Hofestädt, Nikolay A. Kolchanov

**Affiliations:** 1 Institute of Cytology and Genetics SB RAS, Novosibirsk, Russia; 2 Novosibirsk State University, Novosibirsk, Russia; 3 Research Centre of Medical Genetics, Moscow, Russia; 4 George Mason University, Fairfax, VA USA; 5 Bielefeld University, Bielefeld, Germany

This thematic issue of *BMC Genomics* continues the series of BioMed Central special post-conference journal issues presenting materials from the bioinformatics and systems biology summits BGRS\SB (Bioinformatics of Genome Regulation and Structure\Systems Biology). BGRS/SB conference held in Novosibirsk biannually. Here we present the studies in the broad field of genomics as they were presented and discussed at Belyaev Conference-2017 (“Belyaev Readings-2017”- http://conf.bionet.nsc.ru/belyaev100/en). This Special Issue is accompanied by the other Special Issues collecting the works in the fields of evolutionary biology, plant biology, and genetics, which were published as *BMC Evol Biol* [1], *BMC Genetics* [2] and *BMC Plant Biol* [3] supplements, respectively.

The Year 2017 marked the 100-th anniversary since Full Member of the USSR Academy of Sciences, Professor Dmitry K. Belyaev (1917–1985) was born. D. K. Belyaev was an outstanding scientist, evolutionist and geneticist. In view of this memorable date, the Institute of Cytology and Genetics of the Siberian Branch of the Russian Academy of Sciences (ICG SB RAS) held international Belyaev Conference on Genetics and Evolution (Novosibirsk, August 7–10, 2017). In 2017 “Vavilov Journal of Selection and Breeding” published memoirs about Prof. Belyaev (http://vavilov.elpub.ru/jour/issue/view/32/showToc), including article by Prof. Shumny [4] who walks the reader along milestones of Belyaev’s life, Other publications discuss critical contribution of Belyaev’s work to the theory of evolution and domestication [5]. Evolutionary aspects of Belyaev’s work were described in Special Issue of *BMC Evol Biol* (https://bmcevolbiol.biomedcentral.com/articles/supplements/volume-17-supplement-2) [1].

The memorial Belyaev conference-2017 continued the traditions of BGRS\SB by keeping several science sections which were run simultaneously.

Previously published special issues of *BMC Genomics, BMC Genetics,* and *BMC Evolutionary Biology* covered the proceeding of BGRS\SB-2016 conference and SBB-2015 School in Novosibirsk [6–10]. First special issue on genomics was presented at the BGRS\SB-2014 conference series in Novosibirsk (https://bmcgenomics.biomedcentral.com/articles/supplements/volume-15-supplement-12). The materials on evolutionary biology and genetics were recently published in *BMC Evol Biol* (https://bmcevolbiol.biomedcentral.com/articles/supplements/volume-17-supplement-2) and *BMC Genetics* Supplements (https://bmcgenet.biomedcentral.com/articles/supplements/volume-18-supplement-1), correspondingly [1, 2].

Additionally, Plant-Gen-2017 event held in Almaty, Kazakhstan (http://primerdigital.com/PlantGen2017/en/) gathered international plant scientists and the Committee members of Plant Biology section of the Belyaev Conference-2017 from Novosibirsk, thus joining the research efforts in plant genomics. The papers discussed at Young Scientists School “Systems Biology and Bioinformatics - 2017” (SBB-2017) (http://conf.bionet.nsc.ru/sbb2017/en/) and “NGS in Medical Genetics” (http://ngs.med-gen.ru/) were collated as well.

Genomics-related works discussed at Belyaev conference-2017 are put together in present issue of *BMC Genomics,* including several articles in applied medical genomics and computational genomics applications.

The work by Elena Pudova [11] and co-authors describes epithelial-mesenchymal transition (EMT) in colorectal cancer. Typically, EMT conversion is accompanied by a decrease in cell-cell cohesion and a profound reorganization of the cytoskeleton. The authors analyzed RNA-Seq data derived from The Cancer Genome Atlas (TCGA) project. Analysis of the correlations between expression levels of individual mRNAs allowed scientists to pinpoint the set of co-expressed genes, which included 21 EMT-transition related genes and a single glycolytic gene, *HK3*. Thus, for the first time, an upregulation of *HK3* was associated with EMT. This observation may be of particular importance as one of the metabolic adaptation for rapid proliferation, survival, and metastases.

Irina Chadaeva and colleagues [12] presented genome-wide analysis of single nucleotide polymorphisms (SNP) that alter the affinity of TATA-binding protein for the TATA boxes within the promoters of human genes. When retrospective clinical data were mined for the biomarkers of changes in physiological indicators of reproductive potential, a total of 126 SNP markers of female reproductive potential were uncovered. These genetic variants, capable of altering the affinity of TBP for gene promoters, were identified with an aid of SNP_TATA_Comparator Web service earlier developed by same group of authors. For example, a combination of 10 candidate gene variants related to thrombosis (e.g., rs563763767) may cause overproduction of coagulation inducers. Notably, pregnant women with Hughes syndrome may encounter a fatal thrombosis event, although at the earliest stages of its development this syndrome may be diagnosed, and resultant fatality may be prevented with an assistance of these polymorphisms.

Nikita Ershov et al. [13] used maternal separation mouse model to study effects of early life stress on genomic landscape of H3K4me3 histone modifications within the cells of the prefrontal cortex. Importantly, the distribution of H3K4me3 showed relatively low variability across all individuals, and only some subtle changes revealed in mice with a stressful puppyhood.

The paper presented by Natalia Stefanova and co-authors [14] continues the series of the studies in animal models, where they showed an association of cerebrovascular dysfunction with the development of Alzheimer’s disease-like pathology. This study was performed in OXYS rats – a strain of senescence-accelerated rats, which simulates a number of key characteristics of sporadic Alzheimer’s. According to RNA-Seq analysis, major alterations in cerebrovascular function of OXYS rats are in the blood vessel development, the VEGF signaling pathway, and the contractility of vascular smooth muscle cell contraction. The changes in expression of the genes functionally associated with the cerebrovascular development in early life may contribute to the pathology of Alzheimer’s disease.

Alexey Moskalev and colleagues [15] describe an effects of fucoxanthin on the extent, or if you will, speed of aging. Same group of authors previously showed that the treatment with the fucoxanthin, one of the most abundant marine carotenoids, increases the lifespan in *Drosophila melanogaster* and *Caenorhabditis elegans*. Fucoxanthin are also the components of the so called “functional foods” which supply the body with the vitamins, and microelements required for its healthy maintenance and for prevention of life-style-related diseases like metabolic syndrome. At the organismal level, fucoxanthin increases the median lifespan and had a positive effect on fecundity, fertility, intestinal barrier function, and nighttime sleep. The authors conclude that life-extending effects of fucoxanthin are associated with differential expression of longevity-associated genes.

Olga Voronina and colleagues [16] presented their research on Burkholderiales order microorganisms infecting respiratory tract of cystic fibrosis patients. At least in part, these microbes determine patients’ functional status. Characterization of the Burkholderia strains from the sputum and the sinuses of cystic fibrosis patients provides crucial insights into epidemiology of Burkholderiales.

The next group of articles presents a variety of novel tools for computational genomics.

Igor Titov and Pavel Vorozheykin [17] analyzed miRNA structure of mirtrons, the microRNAs located in the introns of the host genes, and intergenic non-mirtrons. MicroRNAs act through both the canonical and the non-canonical pathways; the most frequently used the non-canonical one is the splicing-dependent biogenesis of mirtrons. The authors compared the mirtrons and non-mirtrons of human and mouse to explore how their maturation shapes up the precursor structure around the miRNA. They noted the coherence of the overhang lengths which indicates the dependence between the cleavage sites. To explain this correlation, they proposed the 2-level model of the Dicer structure that couples the imprecision in either Drosha or Dicer action together. Considering the secondary structure of all animal pre-miRNAs, they confirmed that single-stranded nucleotides are predominantly found near the miRNA boundaries and in its center, which are having much higher mutation rate.

Evgeny Tiys and colleagues [18] present their software FunGeneNet which estimates enrichment of functional interactions within the sets of genes. FunGeneNet utilizes the ANDSystem and STRING to reconstruct gene networks using experimental gene sets and to estimate their difference from random networks. Using examples of thyroid cancer and apoptosis networks, authors have shown that the study of the links over-represented in the analyzed network as compared to random ones aid in biological interpretation.

Fedor Naumenko et al. [19] compare ability of aligner programs to estimate mapping quality and to detect possible sequencing artefacts. They have shown that the validation runs on artificial data improves both accuracy and reliability of peak callers and aligners, while reducing computational time. The authors demonstrate that commonly used statistical methods are insufficient for proper evaluation of aligner performance, and introduce a read density distribution similarity as a novel measure capable of revealing artefacts in aligners’ performances. The proposed quality assessment method allows identification of the inherent shortcoming of aligners not detected by conventional statistical methods.

Elena Salina and co-authors [20] analyzed various genomics features of the bread wheat chromosome 5BS. A physical map of the 5BS arm (290 Mbp) was constructed using restriction fingerprinting and LTC software for contig assembly of BAC clones. After anchoring the BAC contigs on the 5BS chromosome, including clone-by-clone screening of BACs, GenomeZipper analysis, and comparison of BAC-fingerprints with in silico fingerprinting of 5B pseudomolecules of *T. dicoccoides* was performed. This work presents various practical considerations for exploring plant chromosome organization.

Follow-on series of related works in the areas of genomics, genetics, and plant biology discussed at “Belyaev conference – 2017” and other related meetings in Novosibirsk are published in the Special Issues of *BMC Evolutionary Biology (*https://bmcevolbiol.biomedcentral.com/articles/supplements/volume-17-supplement-2)*, BMC Plant Biology* (https://bmcplantbiol.biomedcentral.com/articles/supplements/volume-17-supplement-2)*, BMC Genetics* (https://bmcgenet.biomedcentral.com/articles/supplements/volume-18-supplement-1) (published at the end of 2017), *BMC Medical Genomics* (https://bmcmedgenomics.biomedcentral.com/volume-11-supplement-1), *BMC Structural Biology* (https://bmcstructbiol.biomedcentral.com/articles/supplements/volume-18-supplement-1) and *BMC Neuroscience* (https://bmcneurosci.biomedcentral.com/articles/supplements/volume-19-supplement-1) (published in parallel to this issue in 2018)*.* The Proceedings of the conference are available at http://conf.bionet.nsc.ru/belyaev100/en/.

The readers are welcome to visit Novosibirsk at the time of next XI-the BGRS\SB-2018 conference on August 20-28th in 2018 (http://conf.bionet.nsc.ru/bgrssb2018/en/).
